# Nonlinear Feature-Based MI Detection Supported by DWT and EMD on ECG: A High-Performance Decision Support Approach

**DOI:** 10.3390/bios16030150

**Published:** 2026-03-04

**Authors:** Ali Narin, Merve Keser

**Affiliations:** Department of Electrical and Electronics Engineering, Zonguldak Bülent Ecevit University, Zonguldak 67100, Türkiye; m.baskaya@fbe.karaelmas.edu.tr

**Keywords:** myocardial infarction, ECG, Lead II, intrinsic mode functions, discrete wavelet transform, non-linear features, particle swarm optimization

## Abstract

Myocardial infarction (MI) is a life-threatening cardiovascular disorder caused by a partial or complete interruption of oxygenated blood flow to the myocardium, leading to high mortality rates if not diagnosed promptly. Although electrocardiogram (ECG) signals are widely used due to their non-invasive and low-cost nature, MI-specific abnormalities may be subtle and subject to inter-observer variability. Therefore, reliable artificial intelligence-based decision support systems are essential to enhance diagnostic classification accuracy. In this study, only the Lead II derivation from 12-lead ECG recordings of 52 healthy individuals and 148 MI patients was analyzed. To effectively characterize the non-stationary nature of ECG signals, a hybrid time–frequency feature extraction framework was employed. Five-level intrinsic mode functions and wavelet detail and approximation coefficients were obtained using Empirical Mode Decomposition and Discrete Wavelet Transform with a Daubechies-6 wavelet. From these components, 390 times, nonlinear and complexity-based features were extracted using 23 entropy-driven measures. Particle Swarm Optimization was applied to select the most discriminative feature subset, significantly enhancing classification performance. The optimized features were evaluated using Support Vector Machines, Artificial Neural Networks, k-Nearest Neighbors, and Bagged Tree classifiers. The Bagged Trees classifier achieved the best classification performance with an overall correct classification rate of 97.6%. The results demonstrate that the proposed hybrid feature representation combined with PSO-based selection provides a robust and reliable framework for MI detection, offering strong potential for clinical decision support applications.

## 1. Introduction

The heart is an organ located in the left side of the chest cavity, composed of highly organized muscle tissue, which is essential for sustaining life. The human heart contracts approximately 100,000 times per day and nearly 40 million times per year, pumping millions of liters of blood into the systemic circulation [1]. This vital organ is susceptible to numerous structural and functional pathologies; heart valve diseases, myocardial disorders, coronary artery blockages (myocardial infarction—MI), and various inflammatory cardiac diseases are among clinically significant examples.

Among these pathologies, MI has the highest clinical significance. MI is defined as a critical cardiovascular event resulting from a severe reduction or complete cessation of coronary blood flow, leading to prolonged myocardial ischemia and subsequent cellular damage [2]. The literature reports that approximately 32% of global deaths are due to cardiovascular diseases, and that 85% of these deaths are due to heart attacks and strokes [3]. Therefore, early detection of possible myocardial infarction and the implementation of preventive approaches are of great importance in minimizing the risk of loss of life in individuals.

The diagnosis of MI is currently performed using multiple clinical methods. These methods include exercise stress testing (EST), cardiac catheterization, and electrocardiogram (ECG) [4,5]. Cardiac catheterization is an invasive procedure requiring advanced expertise and specialized training; during the procedure, patients may face risks such as procedural complications, radiation exposure, and potential nephrotoxicity due to the use of contrast agents, albeit at a low rate [6]. During the EST process, ECG recordings are taken during treadmill exercise, and this test also carries a rare risk of cardiac arrest [7]. Therefore, not every MI patient may be a suitable candidate for EST.

Electrocardiography (ECG) is a non-invasive diagnostic technique that records the electrical activity of the heart using surface electrodes placed on the body, reflecting cardiac depolarization and repolarization processes over time [8]. ECG signals play a critical role in the early diagnosis of MI due to their rapid acquisition, low cost, and widespread clinical availability [9]. Although ECG recordings are routinely interpreted by expert clinicians, the complex morphology of cardiac waveforms, low-amplitude variations, and inter-patient electrophysiological differences may lead to misinterpretations in conventional visual assessment [10]. With advances in information technology, automated ECG analysis and computer-aided decision support systems have increasingly been adopted to reduce observer-dependent variability and support clinical decision-making [11].

In the literature, numerous methods have been developed for the automatic detection and localization of MI using ECG data. These approaches are generally classified into three main categories: traditional methods, machine learning (ML)-based models, and deep learning (DL) techniques. Traditional methods rely on clinicians manually interpreting changes in the ECG signal, particularly elevation or depression in the ST segment [12], morphological alterations in the T and Q waves [13], and changes in the PR and QT intervals [14]. However, the inherently subjective nature of these methods and the variability associated with the interpreter’s experience pose a significant limitation.

In recent years, machine learning (ML)-based approaches, defined as data-driven computational models capable of learning patterns and making predictions, have gained increasing attention in ECG signal analysis. In this context, methods such as Discrete Wavelet Transform (DWT) and Empirical Mode Decomposition (EMD) are widely used to increase the time–frequency resolution of signals [15,16,17]. While wavelet-based analyses reveal the multi-resolution structure of the signal, enabling more effective capture of MI indicators in low-frequency components, EMD highlights the local characteristics of the signal through Intrinsic Mode Function (IMF) components obtained adaptively [15,18].

In the feature extraction process, there has been growing interest in the use of entropy-based measures to quantitatively express the irregularity and complexity of the signal. Methods such as energy entropy, sample entropy, and Rényi entropy have been reported to successfully characterize the chaotic and nonlinear structure of ECG signals [19,20]. These extracted features have been classified using support vector machines (SVMs), artificial neural networks (ANNs), k-nearest neighbors (k-NNs), and various ensemble learning algorithms; classification accuracy rates above 90% have been reported in many studies [21,22,23,24].

However, higher classification performance has been achieved in myocardial infarction detection using advanced machine learning and deep learning approaches [25,26,27]. In particular, one-dimensional convolutional neural networks (1D-CNNs) provide high-accuracy classification performance without requiring a traditional feature extraction step by learning directly from the raw ECG signal [28,29]. Furthermore, time-dependent models such as long short-term memory (LSTM) networks have achieved improved diagnostic classification performance by capturing temporal dependencies inherent in ECG signals [30,31]. These studies report model classification accuracies mostly above 95%.

All these findings indicate that ECG-based MI diagnosis systems are becoming increasingly automated and have reached a level of maturity where they can be integrated into clinical decision support systems. However, due to the high amount of labeled data required by DL models, the importance of ML-based methods that can perform well even on smaller datasets remains; supported by hybrid approaches, their potential for clinical use is increasing.

Early and accurate diagnosis of MI is essential for reducing mortality and morbidity associated with cardiovascular diseases. In recent years, reliable MI detection using single-lead ECG signals has attracted increasing interest due to its suitability for portable monitoring systems and low-cost early warning applications. Although multi-lead ECG configurations provide comprehensive cardiac information, they often increase hardware complexity and computational burden, limiting their applicability in real-time and wearable healthcare systems. Consequently, developing efficient diagnostic approaches based on single-lead ECG recordings remains an important research challenge.

However, ECG signals exhibit nonlinear, nonstationary, and noise-sensitive characteristics that restrict the effectiveness of conventional linear analysis methods. To better capture these complex dynamics, advanced signal decomposition and nonlinear feature representation techniques have been increasingly explored. In this study, denoised ECG signals were analyzed using DWT and EMD, enabling multi-level characterization of cardiac activity across temporal and frequency domains. Statistical and entropy-based measures were extracted from the resulting components to construct a comprehensive feature representation reflecting signal complexity.

**Figure 1:**
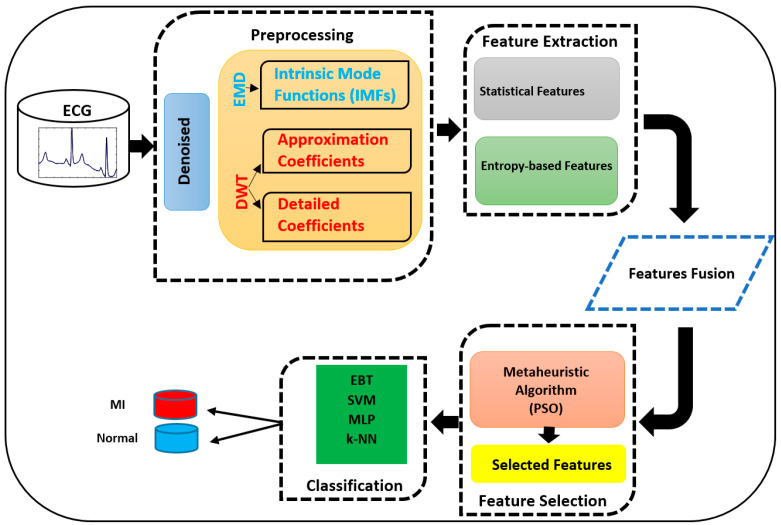
Overall workflow of the myocardial infarction detection framework employed in this study.

The overall analysis framework (Figure 1) integrates preprocessing, multi-level decomposition, nonlinear feature extraction, particle swarm optimization (PSO)-based feature selection, and comparative machine learning classification. By jointly analyzing wavelet coefficients and intrinsic mode functions, the proposed framework enables systematic investigation of component-level contributions to MI detection performance. The optimized feature subsets were evaluated using multiple classifiers, including SVM, ANN, k-NN, and ensemble-based BT.

Through this integrated decomposition–optimization–classification strategy, high diagnostic classification performance was achieved using only Lead II ECG signals. The findings demonstrate that accurate MI detection can be achieved with reduced acquisition complexity, highlighting the practical potential of the proposed framework for lightweight and clinically deployable decision-support systems.

The remainder of the study presents the data set introduction, preprocessing steps, details of the DWT and EMD methods, feature extraction processes, feature selection algorithm, classification models, performance metrics, experimental findings, and discussion sections.

## 2. Material and Methods

### 2.1. Data Set

In this study, the Physikalisch-Technische Bundesanstalt (PTB) database, publicly available on the PhysioNet.org website, was used [32]. The database contains a total of 549 standard clinical 12-lead ECG recordings obtained from 290 individuals (209 male and 81 female). Clinical information was unavailable for 22 recordings; therefore, these samples were excluded from the study. There are 52 recordings from healthy individuals and 148 recordings from MI individuals, which are the subjects of this study. Each signal was sampled at a resolution of 16 bits and a sampling rate of 1000 Hz.

In this study, all records belonging to healthy individuals and those diagnosed with myocardial infarction in the ECG database were evaluated. Analyses were performed specifically on Lead II derivation, which is widely used in systems such as long-term monitoring (Holter monitoring), mobile ECG devices, and smart watches/devices, which are the most frequently preferred in hospital and ambulance settings [33,34]. As shown in Table 1, each ECG recording, originally consisting of signals with varying lengths, was segmented into fixed-length portions of 10,000 samples, where each sample represents a single recorded ECG data point in the time domain. Using this segmentation procedure, a total of 928 healthy and 3934 MI signal segments were obtained and used for subsequent analysis.

All ECG signals were denoised using DWT implemented in MATLAB 2023a with the Wavelet Toolbox. A Daubechies-6 (db6) mother wavelet with six-level decomposition was applied, and noise components were suppressed through thresholding of detail coefficients followed by inverse DWT reconstruction [35].

Prior to feature extraction, all denoised ECG signals were normalized according to the formulation given in Equation (1).

(1) $$X_{norm}=\frac{\left(X-X_{min}\right)}{X_{max}-X_{min}}$$

where X is the original signal; $X_{min}$ and $X_{max}$ respectively represent the minimum and maximum values of the signal. The main reason for choosing min–max normalization is that this method preserves signal morphology and is relatively robust against outliers.

**Figure 2:**
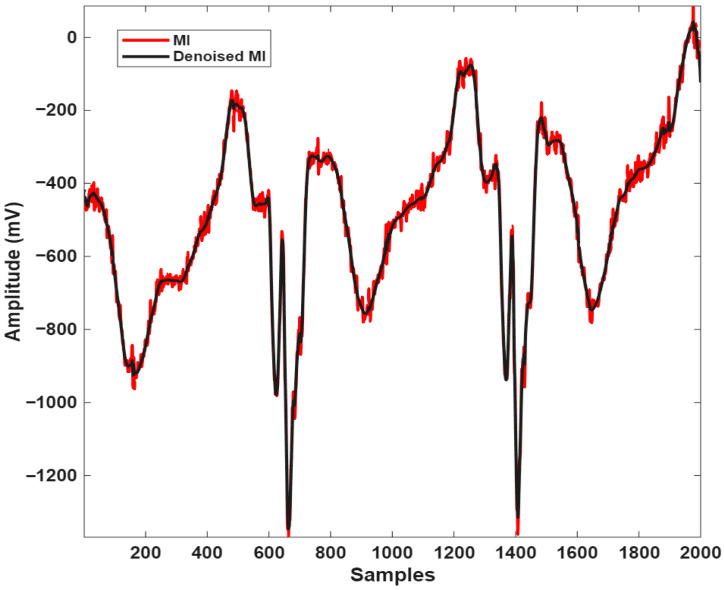
Overlap of noisy (red) and noise-free (black) ECG signals belonging to the MI individual.

**Figure 3:**
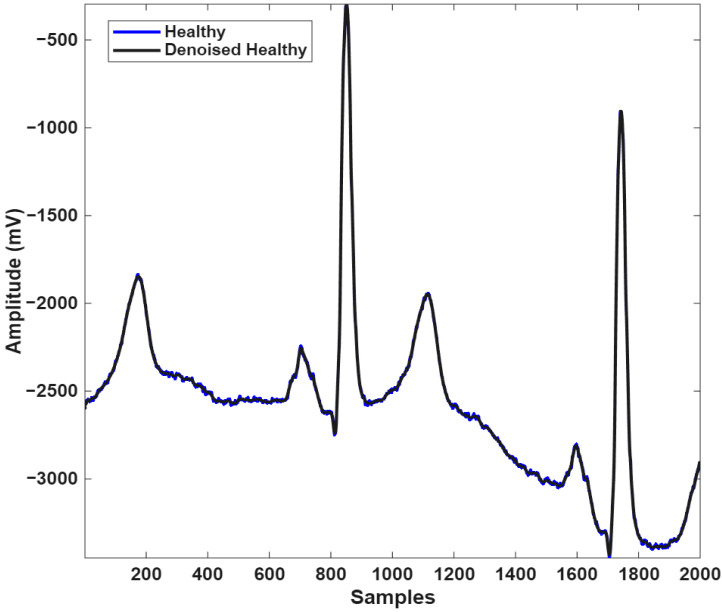
Overlap of noisy (blue) and noise-free (black) ECG signals from a healthy individual.

Figure 2 and Figure 3 present the original ECG signals of healthy individuals and MI patients along with the corresponding denoised signals obtained through DWT-based noise reduction.

### 2.2. Discrete Wavelet Transform

The DWT, one of the time and frequency domain transform analyses, enables high-resolution analysis of signals. In this method, signals are decomposed into their frequency components using high-pass and low-pass filters. The output of the low-pass filter is called the approximation coefficients, representing the low-frequency components of the signal, while the output of the high-pass filter is called the detail coefficients, representing the high-frequency components of the signal [36,37].

**Figure 4:**
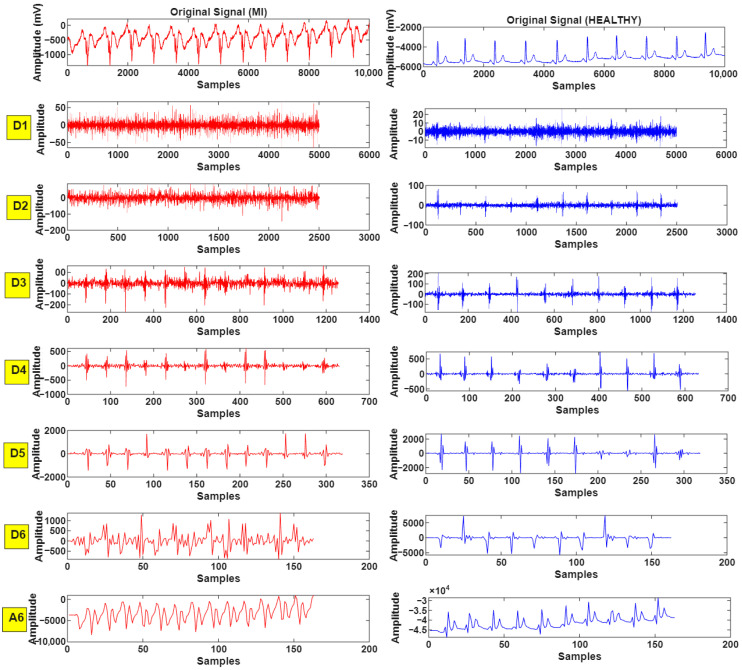
Detail (D1–D6) and approximation (A6) coefficients derived from six-level DWT decomposition of ECG signals for healthy and MI subjects.

In this study, a six-level discrete wavelet transform (DWT) was applied to ECG signals using MATLAB and the Wavelet Toolbox with a Daubechies-6 (db6) mother wavelet. The resulting detail and approximation coefficients obtained at each decomposition level were subsequently used for feature extraction and analysis. This transform preserves both frequency and time information, enabling the acquisition of smoother coefficients that better reflect the original structure of the signal (Figure 4).

The general mathematical expression of the discrete wavelet transform is given below:

(2) $$ADD\left(m,n\right)\;=\;2^{\frac{-m}{2}}\int y\left(t\right)\cdot \psi \left(2^{-m}\cdot t-n\right)dt$$

where y(t) denotes the ECG signal, ψ is the mother wavelet, m represents the decomposition scale, n is the translation parameter, and ADD(m,n) corresponds to the resulting wavelet coefficients.

Figure 4 shows the six-level coefficients for the ECG signals of a healthy person and a person with MI.

### 2.3. Empirical Mode Decomposition

EMD is an adaptive signal processing technique widely used for analyzing nonlinear and non-stationary signals by decomposing them into IMFs representing oscillatory components at different frequency scales. In this study, EMD was implemented in MATLAB using built-in signal processing functions to decompose denoised ECG segments into IMF components arranged from high- to low-frequency content. The decomposition was performed using the standard sifting procedure, in which local maxima and minima were identified, and upper and lower envelopes were generated via cubic spline interpolation. The mean envelope was subsequently removed from the signal, and the process was iteratively repeated until the resulting component satisfied IMF conditions, namely (i) the number of zero crossings and extrema differed at most by one, and (ii) the envelopes defined by local extrema were symmetrically distributed around the signal. Following decomposition, five IMF components obtained from each ECG segment were used for subsequent feature extraction and analysis. The mathematical representation of the Hilbert transform is as follows [38,39]:

(3) $$y\left(t\right)\;=\;H\left[x\left(t\right)\right]\;=\;\frac{1}{\pi }\int_{-\infty }^{\infty }\frac{x\left(\tau \right)}{t-\tau }d\tau$$

where x(t) denotes the ECG signal, y(t) is the transformed signal, H[⋅] represents the Hilbert operator, and t and τ correspond to time and time-shift variables, respectively.

**Figure 5:**
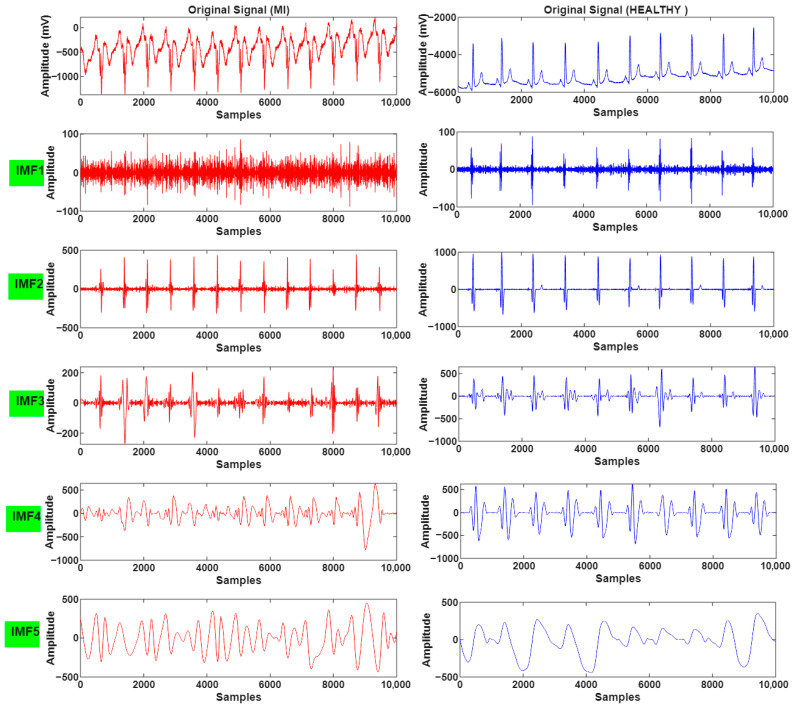
IMF components (IMF1–IMF5) derived from EMD of ECG signals for MI and healthy subjects.

The decomposition results of the obtained IMF components are presented in Figure 5 for both classes.

This separation enabled the detailed characterization of the time–frequency structures of the signals and made it possible to extract meaningful features that can be used to distinguish between pathological and normal conditions. Thus, features with higher discriminative power have been provided to classification algorithms.

### 2.4. Feature Extraction

In this section, feature extraction was conducted using DWT-derived detail and approximation coefficients from denoised ECG segments, along with five-level IMF components obtained through EMD. Time-domain and nonlinear features were extracted from these data. Details of the extracted features are presented in the following sections.

#### 2.4.1. Time Domain Measurements

Time domain-based statistical features were included in the analysis process to determine the fundamental structural characteristics of the ECG signals. These features are numerical measures that directly represent the amplitude, variation, and distribution characteristics of the signal in the time domain. They are frequently preferred to provide information about the basic trends and waveforms of physiological signals [40,41].

The time domain features used in this study were as follows: Minimum, maximum, mean, variance, root mean square (RMS), skewness, and kurtosis. Minimum represents the smallest value of the signal. Maximum represents the largest value of the signal. Mean reflects the general level trend of the signal. Variance provides information about the signal’s energy and stability by showing the level of deviation of the samples from the mean. RMS is particularly important for periodic structures, reflecting the average power value dependent on the signal’s amplitude. Skewness determines whether the signal distribution is symmetric, revealing asymmetries in the positive or negative direction. Kurtosis analyzes whether the signal is concentrated around the center by providing information about the sharpness of the distribution’s peak and the frequency of extreme values.

These features are fundamental metrics evaluated within the scope of linear analysis and reveal the general structural characteristics of the signal at a low computational cost. However, since these metrics cannot fully reflect the complex and multi-scale structure of the signal, they have been used in a complementary manner with entropy-based nonlinear features in this study.

#### 2.4.2. Entropy-Based Feature Extraction

ECG signals are inherently irregular and exhibit complex structures with time-dependent variability. Therefore, the use of nonlinear analysis techniques is becoming increasingly common, especially in the detection of cardiac abnormalities such as MI. Time, frequency, and time–frequency domain analyses may be insufficient in identifying natural variations in the ECG; however, nonlinear methods enable the characteristic structural features of the signals to be revealed more effectively.

The use of nonlinear techniques in feature extraction from components obtained via the EMD method increases computational costs but enables the acquisition of more meaningful and discriminative features [42]. In this context, one of the most effective methods used to quantitatively assess signal complexity is entropy analysis.

Entropy is a complexity metric that measures irregularity, randomness, and information density of time series. The structural order in physiological signals is disrupted by factors such as disease and aging, leading to significant differences in entropy levels. ECG signals from healthy individuals are generally characterized by higher entropy and complexity values, as they possess more regular and effective physiological communication mechanisms. This reflects the level of interconnection and coordination of the underlying biological systems.

In this study, a total of 23 different entropy-based features, defined in the literature and representing different analysis approaches, were extracted to comprehensively represent the nonlinear characteristics of ECG signals. The entropy types used include Attention, Conditional, Cosine Similarity, Distribution, Entropy of Entropy, Grid Distribution, Increment, Phase, Slope, Spectral, Symbolic Dynamics, Tsallis, Renyi, Wavelet, Hurst Exponent, Fuzzy, Hierarchical Multiscale, Kolmogorov–Sinai, Multiscale, Permutation, Enhanced Multiscale, and Sampling entropies. These entropy metrics characterize the amplitude, frequency content, structural irregularity, and multiscale dynamics of signals in a multidimensional manner. The theoretical definitions and calculation details for each entropy type are presented in the relevant literature [43,44,45,46,47,48,49,50,51,52].

The nonlinearity addressed in this study relates to signal analysis and feature extraction rather than the physical measurement characteristics of ECG sensors. Accordingly, the proposed approach does not introduce measurement-scale nonlinearity but aims to better represent the inherent nonlinear dynamics of cardiac activity for improved classification performance.

### 2.5. Feature Selection with PSO

In this study, feature selection was applied to improve classification performance and reduce computational load. The initial set of 390 features consisted of time domain, frequency domain, time–frequency analysis, and entropy-based nonlinear complexity measurements. However, it is known that not all features in this high-dimensional feature set contribute equally to classification performance. Therefore, the PSO algorithm was used to determine the optimal feature subset. Although the feature extraction process reduces ECG signals to a more manageable representation space, it does not guarantee that all extracted features will be equally informative or discriminative for the classifier. Unnecessary or noise-sensitive features can increase model complexity, raise computational costs, and trigger the risk of overfitting, especially in limited datasets. To overcome these methodological challenges and systematically identify the feature subset that will provide the highest classification performance, the meta-heuristic optimization method PSO was preferred in this study.

PSO is a population-based stochastic optimization algorithm inspired by the collective intelligence behavior of social organisms [53,54]. The fundamental principle of the algorithm is based on the simulation of the behavior of particles that move within the solution space and share information with each other. In PSO, each particle (*i*, *p*) is represented by a position vector (*xᵢ*(*t*)) and a velocity vector (*vᵢ*(*t*)). To reach the best solution, particles iteratively update their positions and velocities according to the following update equations, referencing their own best experience (*pbest_i_*) and the best experience in the swarm (*gbest*). The mathematical representation is given in Equations (4) and (5).

(4) $$v_{i}^{t+1}=\omega v_{i}^{t}+c_{1}r_{1}\left(pbest_{i}-x_{i}^{t}\right)+c_{2}r_{2}(gbest-x_{i}^{t})$$

(5) $$x_{i}^{t+1}=x_{i}^{t}+v_{i}^{t+1}$$

where *ω* represents the inertia weight, *c*_1_ and *c*_2_ represent the cognitive and social learning coefficients, respectively, and *r*_1_ and *r*_2_ represent random numbers uniformly distributed in the range [0, 1], respectively. The detailed pseudocode of the PSO algorithm is presented in Algorithm 1, which summarizes the sequential steps used for feature subset optimization in this study. The fitness function used to measure the search performance of the algorithm is structured to maximize classification accuracy while minimizing the number of selected features, thereby increasing model simplicity and computational efficiency.
**Algorithm 1** Pseudocode of the PSO-based feature selection procedure used to determine the optimal subset of discriminative ECG features.**Input:** Feature matrix (*X*), Class labels (*y*)
**Output:** Optimal feature subset (gBest)
Procedure PSO_Feature_Selection()
    Initialize the particle population with random binary feature subsets
    Initialize particle velocities and PSO parameters (*ω*, *c*_1_, *c*_2_)
    **while** termination condition not met **do**
        **for** each particle in the population **do**
            Evaluate fitness using selected features (e.g., classification accuracy)
            Update personal best position (pBest) and global best (gBest)
            Update velocity and position using standard PSO equations
            Apply sigmoid transfer function to binarize new positions
        **end for**
        Update global best solution if needed
    **end while**
Return: gBest → selected optimal feature subset

### 2.6. Classification Algorithms

#### 2.6.1. Bagged Trees

The ensemble learning method aims to create a powerful classifier by combining multiple weak learners (e.g., decision trees) [55]. This approach makes decisions by combining the outputs of different models and generally provides higher classification accuracy and better generalization compared to individual classifiers. The most common ensemble methods include bagging, boosting, and random forest.

The Bagged Trees algorithm used in this study consists of multiple decision trees trained on subsets of the training data created using bootstrap sampling. Each tree is trained independently, and classification is performed by majority vote. This reduces the overfitting problem exhibited by individual decision trees and increases the stability of the model [56,57].

#### 2.6.2. Support Vector Machines

SVMs are a supervised learning algorithm developed based on statistical learning theory and exhibit high performance in both linear and non-linear problems [58]. The fundamental principle of SVM is to determine an optimal hyperplane that separates the two classes in the feature space and maximizes the margin. The algorithm creates two parallel boundary hyperplanes using support vectors, which are the closest data points belonging to each class. When the distance (margin) between these hyperplanes is maximized, an optimal discriminant hyperplane with high generalization ability is obtained.

For linearly inseparable data sets, SVM nonlinearly transforms the data into a higher-dimensional feature space using kernel functions. This method allows nonlinear boundaries in the original feature space to be expressed as a linear hyperplane in the high-dimensional space. Commonly used kernel functions include the Gaussian kernel (RBF), polynomial kernel, and sigmoid kernel. This flexibility enables SVM to demonstrate effective classification performance, particularly on datasets with complex structures.

#### 2.6.3. Artificial Neural Networks

ANNs are computational models inspired by the functioning of biological neural systems, consisting of artificial neurons capable of parallel processing. These models typically consist of an input layer, one or more hidden layers, and an output layer. In the system, feature vectors applied to the input layer are transmitted to successive layers with adjustable weight coefficients. Each hidden layer neuron calculates the weighted sum of the inputs it receives to form a net activation value, and this value is converted to output by passing through a nonlinear activation function [59].

During the training process of ANNs, weight updates are performed using the backpropagation algorithm until the error between the network outputs and the target values is minimized. Therefore, one of the most critical factors determining the generalization performance of the model is the information content and representational power of the input feature vectors. Inappropriately selected features can significantly reduce the classification accuracy of the model.

In this study, all extracted features and selected features were fed as input data to the ANN model.

#### 2.6.4. k-Nearest Neighbor

k-NN is a lazy learning-based classification algorithm that is frequently preferred due to its simple structure and effective performance [60]. It does not require model creation; only attributes and class information are stored during the training phase.

Each example is considered a point in d-dimensional space. An example with an unknown class is classified according to the class majority of its k-nearest neighbors based on their distances to the examples in the training data. The k value is usually small and unique, which prevents indecisiveness. Distance measures such as Euclidean or Mahalanobis can be used.

k-NN does not make assumptions about data distribution and has high generalization potential. Storing training data prevents information loss. However, it can be slow with large datasets and may be affected by noise. Therefore, feature scaling is recommended.

### 2.7. Performance Metrics

This section describes some basic performance metrics used to evaluate the performance of different classifiers used in the study. These metrics are accepted by experts and are used to evaluate the effectiveness of automatic diagnosis systems [61].

**Figure 6:**
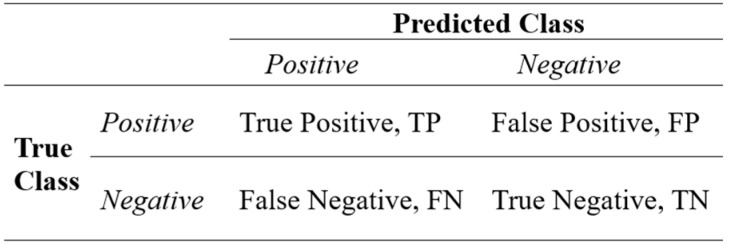
Structure of the confusion matrix used for performance evaluation, illustrating the relationship between true and predicted classes.

Classification performance is evaluated by comparing actual and predicted classes based on the accuracy of positive and negative decisions. In this context, four scenarios emerge based on actual and predicted results: true positive (TP), false positive (FP), true negative (TN), and false negative (FN). In this study, individuals with myocardial infarction (MI) were considered positive, while healthy individuals were considered negative. If individuals with actual MI are correctly classified as MI, they are considered TP; if they are classified as healthy, they are considered FN. If healthy individuals are correctly classified as healthy, they are considered TN; if they are classified as MI, they are considered FP. These four scenarios can be represented using a 2 × 2 decision matrix (Figure 6).

The basic criteria used in performance evaluation are as follows: Accuracy (Acc), Recall (Rec), Specificity (Spe), Positive Predictive Value (PPV), and Negative Predictive Value (NPV). These criteria are calculated as follows [62]:

(6) $$Acc=\frac{TP+TN}{TP+TN+FP+FN}$$

(7) $$Rec=\frac{TP}{TP+FN}$$

(8) $$Spe=\frac{TN}{TN+FP}$$

(9) $$PPV=\frac{TP}{TP+FP}$$

(10) $$NPV=\frac{TN}{TN+FN}$$

The classification accuracy of performance results also depends on how the training and test datasets are separated. In the literature, this separation is typically performed using k-fold cross-validation or “leave-one-out” cross-validation methods. In this study, results were obtained using the 10-fold cross-validation method.

All signal processing, feature extraction, optimization, and classification analyses were conducted in MATLAB on a computer equipped with an Intel i5-8265U processor (2.5 GHz) and 8 GB RAM.

## 3. Experimental Results

In this study, ECG recordings from the PTB database, publicly available on the PhysioNet platform, were used to ensure the reliability and scalability of the methods. The dataset contains signals from 52 healthy individuals and 148 myocardial infarction patients.

All data were denoised using DWT and then scaled using the min–max normalization method. The total data length was recorded as 3,934,000 samples in the myocardial infarction (MI) group and 928,000 samples in healthy individuals. These signals were segmented into segments, each 10,000 samples long, yielding 3934 MI and 928 healthy data samples.

Using all this data, detail and approximation coefficients were extracted for each signal by applying a 6-level DWT. Additionally, IMFs were obtained using a 5-level EMD method on the original signals, creating a total of 13 different signal groups, including the original signal.

A total of 30 features were extracted for each signal group, including time-domain-based statistical measurements (minimum, maximum, mean, variance, RMS, skewness, and kurtosis) and 23 different entropy types. The classification performance of these features was analyzed separately using the BTs, SVMs, ANNs, and k-NNs algorithms, and the results obtained are presented in detail in the relevant sections. Furthermore, the obtained features were selected using the PSO algorithm, and the classification performance of these selected features was also evaluated.

### 3.1. Effect of Discrete Wavelet Transform Method on Myocardial Infarction Detection

This section examines the effects of DWT applied to denoised signals at different levels and their impact on classification performance. Each transformation coefficient was evaluated separately, and then a holistic scenario using all detail and approach coefficients together was analyzed. The findings are summarized in Table 2.

It can be clearly seen in Table 2 that the original dataset demonstrated strong performance at the initial level with the classification accuracy rate of approximately 94.7–94.8%. The D1 and A6 coefficients provided lower classification accuracy, remaining in the range of 84.5–87.6%. However, the D4 coefficient in particular showed high discriminative power on its own, yielding quite successful results in the range of 93.6–95.4% classification accuracy in both traditional (BT, SVM) and example-based (k-NN) algorithms. This indicates that the D4 coefficient strongly represents the characteristic features of the signals.

The highest classification accuracy was achieved in all A and D datasets, where all detail and approach coefficients were used together. In this scenario, the BT algorithm was the most successful method with 97.2%, followed by ANN with 96.3% and SVM with 95.8% classification accuracy rates. The results reveal that increasing the number of features and evaluating different coefficients together significantly improves classification performance. Overall, the BT and ANN models demonstrated more consistent and higher performance compared to other classifiers.

### 3.2. Effect of the Experimental Mode Decomposition Method on MI Detection

This section examines the effect of IMFs obtained from ECG signals on classification performance. The classification accuracy values obtained by evaluating the features extracted from each IMF using different classification algorithms are presented in Table 3.

The results reported in Table 3 clearly indicate that the classification accuracy rates gradually decrease as the IMF order increases when IMF components are used individually. This situation reveals that the first IMFs, in particular, represent more information content in the signal. On the other hand, in the all IMFs scenario, where all IMF components are evaluated together, higher classification performances were achieved compared to the original signal. In this context, the k-NN algorithm was the most successful method with a 95.8% classification accuracy rate. This finding shows that using IMF components together more effectively represents the complex structure of the signals and improves classification performance.

### 3.3. Performance of All Features in MI

**Figure 7:**
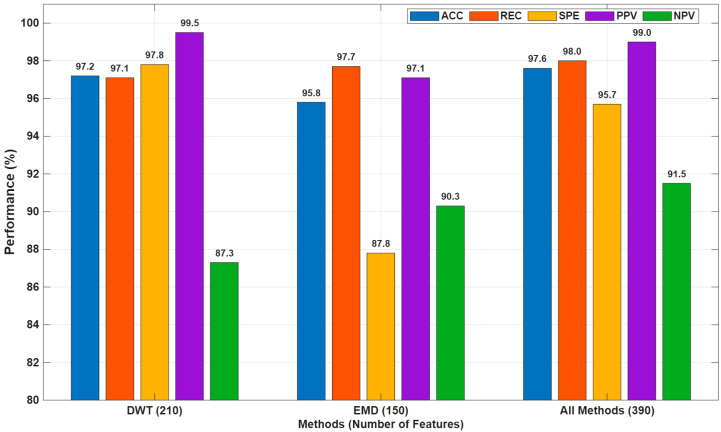
Performance comparison of classification results obtained using DWT, EMD, and all feature sets based on ACC, REC, SPE, PPV, and NPV metrics (%).

The 30 features obtained from the original ECG data, the 210 features extracted using detail and approximation parameters, and the 150 features obtained from the inner mode functions as a result of the experimental mode decomposition method were analyzed together in a comprehensive evaluation. This comparative analysis, along with other results for the BT algorithm, is presented in Figure 7.

As clearly shown in Figure 7, DWT-based features yielded extremely successful results, particularly in the accurate classification of myocardial infarction (MI) individuals, with the highest specificity (97.8%) and positive predictive value (99.5%). This success can be attributed to the sensitivity of the wavelet transform to sudden changes and local frequency components. On the other hand, EMD-based features demonstrated that they could effectively model low-frequency variations and structural features in the signal with the highest sensitivity (97.7%) and negative predictive value (90.3%).

The model obtained by combining DWT and EMD methods exhibited the strongest classification performance with overall classification accuracy (97.6%), sensitivity (98.0%), and balanced specificity (95.7%) values. This demonstrates that different feature extraction methods provide complementary information and that their combined use improves classification performance.

In conclusion, multi-modal feature extraction is a highly effective approach, particularly for complex biomedical problems such as MI detection. Rather than being limited to a single method, hybrid approaches that integrate different representations of information, as in this study, enable the development of more robust and generalizable classification systems. However, using all methods together increased the number of features to 390, potentially increasing computational cost and introducing the risk of overfitting. Therefore, the next section will analyze in detail the impact of PSO-based feature selection on classification performance.

### 3.4. Performance Analysis of PSO-Based Feature Selection in MI Detection

In this section, a comprehensive analysis was conducted to reduce computational cost through feature dimensionality reduction. Decreasing the number of extracted features significantly lowers computational complexity and training time while maintaining classification performance, which is particularly important for real-time and resource-constrained diagnostic applications. In this regard, a binary particle swarm optimization algorithm was employed for feature selection to determine the optimal subset of discriminative features. The PSO-selected features and the corresponding performance results of the BT algorithm are presented in Table 4.

Table 4 shows that feature selection performed using the PSO algorithm significantly reduces the feature size without causing a noticeable decrease in classification performance.

First, the values of 97.2% Acc, 97.1% Rec, 97.8% Spe, and 99.5% PPV obtained using 210 DWT-based features demonstrate that the method offers quite successful performance in detecting MI. Although the number of features decreased to 106 after the PSO application, the classification accuracy decreased only by 0.6% to 96.6%; in the Rec, Spe, and PPV metrics, only very limited decreases were observed, with values of 96.5%, 97.0%, and 99.4%, respectively. This situation reveals that PSO successfully eliminates features with low information contribution in terms of classification, creating a simpler and more efficient model structure.

Similarly, when EMD-based features were used, the initial classification accuracy was 95.8%, but after PSO, when the feature size was reduced from 150 to 65, the Acc dropped to 95.0%. Additionally, partial decreases were observed in metrics directly affecting classification performance, such as Rec (97.7% → 96.2%) and NPV (90.3% → 83.6%). These results show that some features obtained with EMD play a critical role in classification and that eliminating these features has a measurable impact on performance.

Finally, in the hybrid (DWT + EMD) approach, the total number of features was reduced from 390 to 196 using PSO, and the classification accuracy rate decreased by only 0.2%, from 97.6% to 97.4%. Similarly, very limited differences were observed in critical metrics such as Rec (98.0% → 97.7%), Spe (95.7% → 95.9%), and PPV (99.0% → 99.1%). These findings clearly demonstrate that PSO performs an effective selection process in the combined feature space, significantly preserving classification performance while reducing model complexity.

**Figure 8:**
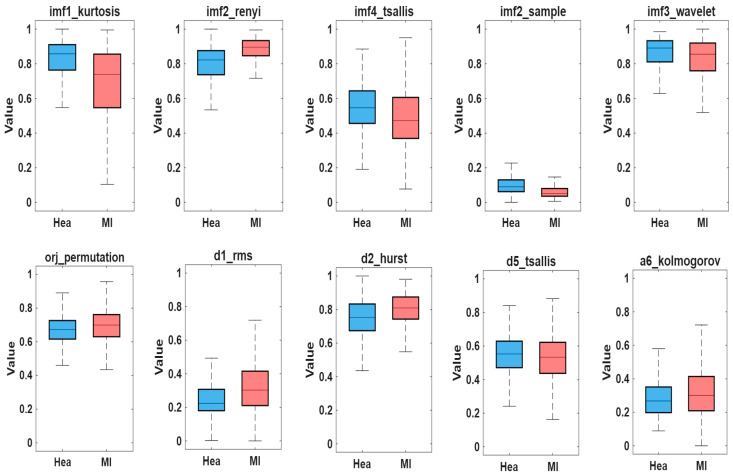
Boxplot distribution of the ten most discriminative features selected by PSO for healthy (Hea) and myocardial infarction (MI) classes. The y-axis represents normalized feature values.

The distribution of the 10 most effective features selected from 196 features using the PSO algorithm is shown in Figure 8 using boxplot graphs. The findings reveal that the selected features provide a clear statistical distinction between the MI and healthy individual (Hea) classes. Specifically, the imf1_kurtosis, imf2_renyi, imf3_wavelet, d2_hurst, and d5_tsallis features show significantly higher median values in the MI class. This trend indicates the effect of irregular cardiac activity during MI on the distribution, entropy, and wavelet components of the ECG signals. The imf4_tsallis, orj_permutation, and a6_kolmogorov features show a more balanced distribution between the two classes; however, the widening of the distribution range in the MI class indicates that the pathological condition increases signal complexity. The imf2_sample and d1_rms features have higher values in healthy individuals, indicating that regular oscillations in normal cardiac rhythms are more pronounced.

In general, the graphs reveal that the vast majority of selected features carry clear discriminative information between the two classes. In particular, the pronounced deviations in distribution medians confirm that the attributes contribute significantly to the classification model. These results show that the attributes selected by PSO play a critical role in MI detection and form an effective basis for improving the model’s overall classification performance.

## 4. Discussion

This study presents an integrated analysis framework that simultaneously targets high classification accuracy, interpretability, and computational efficiency for MI detection based on signals obtained from the Lead II ECG derivation, which is widely used in hospital and ambulance settings. Both classical approaches based on handcrafted features and deep learning-based automatic feature extraction strategies for MI detection have been widely reported in the literature. However, both approaches have limitations: manual features cannot fully represent the nonlinear and non-stationary nature of ECG signals, while deep learning models pose difficulties in clinical integration due to high data and hardware requirements. This study provides original contributions to the literature by presenting a hybrid approach that bridges the gap between these methods.

The findings reveal that the combined use of time–frequency transformations and nonlinear chaotic descriptors accurately represents the morphological variations in MI. Deriving sub-bands obtained with DWT and EMD from entropy-based measurements captured the micro-level complexity and irregularity components of the signals; in this respect, it provided superior discrimination capability compared to classical time/frequency domain features. Indeed, the fact that features derived from DWT Level-4 detail coefficients alone provided 95.4% classification accuracy, and features derived from the EMD IMF-1 component provided 92.8% accuracy, supports that MI representation is largely hidden in the signal’s low-amplitude but high-information frequency components.

Classification experiments conducted on a high-dimensional dataset consisting of a total of 390 features clearly demonstrated that boosting-based ensemble methods exhibit superior performance in complex feature spaces. The classification accuracy, sensitivity, and specificity values of 97.6%, 98.0%, and 95.7%, respectively, offer significantly higher results than classical machine learning studies such as [63,64], as presented in Table 5. It reached similar levels to deep learning methods such as those by [65,66,67]. The most important difference is that the proposed method significantly reduces computational cost and hardware requirements while providing high interpretability. Thus, the results show that well-designed handcrafted feature sets are still competitive at the clinical scale, rather than automatic feature extraction.

During the feature selection phase, PSO-based dimensionality reduction resulted in a relatively small decrease in model classification accuracy (0.2–0.8%). This indicates that some low-importance features—particularly those weighted by entropy and chaotic dynamics—contribute to the model’s overall performance. Therefore, in the future, evaluating methods that provide multi-objective meta-heuristic optimization and stable subset selection could further strengthen the model’s generalizability.

Another critical aspect of this study is that it was evaluated on a large-scale dataset comprising 928 normal and 3934 MI samples, unlike the relatively small datasets used in many studies in the literature. The consistent high performance of the classifiers on a large and heterogeneous sample supports the method’s lack of overfitting tendency and its transferability to real clinical applications.

ECG signals may be influenced by sensor-related factors such as electrode placement variability, motion artifacts, and environmental noise. These measurement uncertainties can affect signal morphology and, consequently, classification performance. While the present study primarily focuses on algorithmic discrimination capability, the robustness of the proposed method against acquisition-related degradations is supported by the use of nonlinear and entropy-based features.

Overall, the findings reveal that the integrated use of nonlinear entropy and complexity measures with time–frequency transformations in MI detection constitutes a powerful alternative to deep learning and offers a viable solution for smart and portable medical diagnostic systems. The model’s high performance, low computational cost, and high interpretability hold significant potential for real-time clinical decision support mechanisms, IoT-enabled ECG analysis devices, and wearable health technologies.

### Limitations of the Study and Future Work

This study has some limitations. First, the dataset used only includes ECG recordings from the Lead II derivation, and the spatial information provided by multi-derivation analysis has not been evaluated in this study. Second, since signal-to-noise levels vary depending on the data source, the model’s performance may vary under different recording conditions (e.g., signals obtained from portable devices). Furthermore, PSO-based feature selection relies solely on a single meta-heuristic algorithm; the impact of different optimization techniques (e.g., Ant Lion, Grey Wolf, or Firefly) on performance has not yet been investigated.

Future studies plan to model spatial-temporal features more comprehensively using multi-lead ECG signals. Furthermore, the integration of the proposed methods into real-time hardware platforms (e.g., portable ECG monitors or embedded systems) is targeted. However, the model’s generalizability will be tested on datasets obtained from different patient groups and clinical settings to evaluate its suitability for clinical applications in greater detail.

## 5. Conclusions

This study presents a hybrid nonlinear feature extraction and optimization framework for automated myocardial infarction detection using single-lead ECG signals. By combining entropy-based complexity features with PSO-driven feature selection, the proposed approach effectively captured discriminative characteristics of nonstationary cardiac signals. Among the evaluated classifiers, the ensemble-based Bagged Trees model achieved the best classification performance, demonstrating the effectiveness of the optimized feature representation.

The findings indicate that the proposed methodology provides a reliable and computationally efficient decision-support framework that may contribute to early MI detection using low-complexity ECG acquisition systems. Future studies will focus on validating the proposed approach using larger and multi-center datasets and investigating robustness under varying signal acquisition conditions.

## Figures and Tables

**Table 1:** Summary of ECG recordings and diagnostic class distribution analyzed using Lead II signals.

Class	Total Record Length	Used Length	Obtained Records
MI	39,340,000	10,000	3934
Normal	928,000	10,000	928

**Table 2:** Comparative classification accuracy (%) of different machine learning classifiers obtained using original ECG signals and DWT-derived detail (D1–D6) and approximation (A6) coefficients based on feature subsets.

Data	Number of Feature	Acc (%)			
		BT	SVM	ANN	k-NN
Original	30	94.7	94.8	94.8	84.2
D1	30	87.6	87.1	86.1	86.1
D2	30	91.0	89.9	89.6	89.4
D3	30	92.7	92.0	92.5	93.9
D4	30	94.4	93.6	94.1	95.4
D5	30	92.0	88.3	89.9	86.3
D6	30	90.3	86.5	88.1	86.9
A6	30	86.5	84.5	85.9	86.1
All A and D	210	97.2	95.8	96.3	94.8

**Table 3:** Comparative classification accuracy (%) of machine learning classifiers using original ECG signals and intrinsic mode function (IMF1–IMF5) components obtained via EMD.

Data	Number of Feature	Acc (%)			
		BT	SVM	ANN	k-NN
Original	30	94.7	94.8	94.8	84.2
IMF1	30	92.8	92.0	92.1	91.9
IMF2	30	89.8	90.1	89.4	90.4
IMF3	30	87.2	87.1	86.9	86.4
IMF4	30	84.3	84.0	82.7	84.2
IMF5	30	83.6	83.5	82.4	82.2
All IMFs	150	94.9	95.2	95.3	95.8

**Table 4:** PSO-Based Feature Selection Before and After MI Classification Performance.

Methods	Number of Features Used and Performance Results (%)						Number of Features Selected with PSO and Performance Results (%)					
	Num. of Fea.	Acc	Rec	Spe	PPV	NPV	Num. of Fea.	Acc	Rec	Spe	PPV	NPV
DWT	210	97.2	97.1	97.8	99.5	87.3	106	96.6	96.5	97.0	99.4	84.7
EMD	150	95.8	97.7	87.8	97.1	90.3	65	95.0	96.2	89.7	97.7	83.6
Orj+DWT+EMD	390	97.6	98.0	95.7	99.0	91.5	196	97.4	97.7	95.9	99.1	90.1

**Table 5:** Literature studies conducted on the detection of MI using the PTB database.

Author/s	Methods	Data	Results
Sadhukhan et al. [63]	DWT, Six features//5-fold//Logistic regression	N: 65 MI: 308	Acc: %95.6 Rec: %96.5 Spe: %92.7
Sopic et al. [68]	Time, Frequency features 72 features//10-fold//Ensemble Random Forest	N: 52 MI: 52	Acc: %82.4 Rec: %87.9 Spe: %78.8
Diker et al. [64]	Morphological, time domain, and discrete wavelet transform properties, 9 features//10-fold//SVM	N: 52 MI: 148	Acc: %87.8 Rec: %86.9 Spe: %88.6
Lui and Chow [65]	Time domains HRV analysis features. 26 features//10-fold//CNN	N: 80 MI: 368	Acc: %92.4 Rec: %97.7
Feng et al. [66]	Feature extraction has not been used. 10 k-fold//CNN	N: 80 MI: 368	Acc: %95.4 Rec: %98.2 Spe: %86.5
Shahnawaz and Dawood [69]	Time–frequency domain, nonlinear features. 23 features//10 k-fold//ANN	N: 52 MI: 148	Acc: %99.1 Rec: %100 Spe: %99.0
Jian [70]	MSN-Net//5-fold CV	N: 52 MI: 148	Acc: %95.7 Rec: %98.0 Spe: %95.7
Sheth et al. [71]	DWT//5 k-fold//CNN	N: 52 MI: 148	Acc: %91.2 Rec: - Spe: -
Sun [67]	FEC-KML//5 k-fold//Multi-channel residual neural networks	N: 52 MI: 148	Acc: %97.7 Rec: %98.6 Spe: %89.5
This study	Time–frequency domain, nonlinear features. 390 features//10-fold//BT	N: 52 (928) MI: 148 (3934)	Acc: %97.6 Rec: %98.0 Spe: %95.7
This study	Time–frequency domain, nonlinear features, PSO, 196 features//10-fold//BT		Acc: %97.4 Rec: %97.7 Spe: %95.9

## Data Availability

The dataset used in this study is publicly available on the PhysioNet.org website [32].

## References

[1] Peirlinck M., Costabal F.S., Yao J., Guccione J.M., Tripathy S., Wang Y., Kuhl E. (2021). Precision medicine in human heart modeling: Perspectives, challenges, and opportunities. Biomech. Model. Mechanobiol..

[2] Lüscher T.F. (2015). Myocardial infarction: Mechanisms, diagnosis, and complications. Eur. Heart J..

[3] World Health Organization Cardiovascular Diseases (CVDs). https://www.who.int/news-room/fact-sheets/detail/cardiovascular-diseases-(cvds).

[4] Unger S.A., Kucia A.M. (2022). Diagnostic procedures. Cardiac Care: A Practical Guide for Nurses.

[5] Li J., Zhao W., Tian Z., Hu Y., Xiang J., Cui M. (2024). Correlation between coronary microvascular dysfunction and cardiorespiratory fitness in patients with ST-segment elevation myocardial infarction. Sci. Rep..

[6] Fernandez R., Ellwood L., Barrett D., Weaver J. (2021). Safety and effectiveness of strategies to reduce radiation exposure to proceduralists performing cardiac catheterization procedures: A systematic review. JBI Evid. Synth..

[7] Höllriegel R., Mangner N., Schuler G., Erbs S. (2013). Physical exercise training and coronary artery disease. Rev. Health Care.

[8] Kligfield P., Gettes L.S., Bailey J.J., Childers R., Deal B.J., Hancock E.W., van Herpen G., Kors J.A., Macfarlane P., Mirvis D.M. (2007). Recommendations for the Standardization and Interpretation of the Electrocardiogram. Circulation.

[9] Thygesen K., Alpert J.S., Jaffe A.S., Chaitman B.R., Bax J.J., Morrow D.A., White H.D. (2018). Fourth Universal Definition of Myocardial Infarction. Circulation.

[10] Salerno S.M., Alguire P.C., Waxman H.S. (2003). Training and competency evaluation for interpretation of 12-lead electrocardiograms: Recommendations from the American College of Physicians. Ann. Intern. Med..

[11] Velandia H., Pardo A., Vera M.I., Vera M. (2025). Systematic Review of Artificial Intelligence and Electrocardiography for Cardiovascular Disease Diagnosis. Bioengineering.

[12] Gong M., Liang D., Xu D., Jin Y., Wang G., Shan P. (2024). Analyzing predictors of in-hospital mortality in patients with acute ST-segment elevation myocardial infarction using an evolved machine learning approach. Comput. Biol. Med..

[13] Han C., Zhou Y., Que W., Li Z., Shi L. (2024). Algorithms for myocardial infarction diagnostics using ECG signals: Advances and challenges. IEEE Trans. Instrum. Meas..

[14] Arif M., Malagore I.A., Afsar F.A. Automatic detection and localization of myocardial infarction using back propagation neural networks. Proceedings of the 4th International Conference on Bioinformatics and Biomedical Engineering.

[15] Zeng W., Yuan C. (2023). Myocardial infarction detection using ITD, DWT and deterministic learning based on ECG signals. Cogn. Neurodyn..

[16] Sahu G., Ray K.C. (2021). An efficient method for detection and localization of myocardial infarction. IEEE Trans. Instrum. Meas..

[17] Zeng W., Shan L., Yuan C., Du S. (2024). Detection of myocardial infarction using Shannon energy envelope, FA-MVEMD and deterministic learning. Complex. Intell. Syst..

[18] Zhou Y., Ma Z., Fu L. (2025). A review of key signal processing techniques for structural health monitoring. Algorithms.

[19] Kumar M., Pachori R.B., Acharya U.R. (2017). Automated diagnosis of myocardial infarction ECG signals using sample entropy. Entropy.

[20] Han C., Shi L. (2019). Automated interpretable detection of myocardial infarction fusing energy entropy and morphological features. Comput. Methods Programs Biomed..

[21] Acharya U.R., Fujita H., Adam M., Oh S.L., Sudarshan V.K., Tan J.H., Koh J.E.W., Hagiwara Y., Chua C.K., Poo C.K. (2017). Automated characterization and classification of coronary artery disease and myocardial infarction by ECG decomposition. Inf. Sci..

[22] Chaitanya M.K., Sharma L.D. (2024). Cross-subject myocardial infarction detection using binary Harris hawks feature selection. IEEE Access.

[23] Siddiqui H.U.R., Zafar K., Saleem A.A., Sehar R., Rustam F., Dudley S., Ashraf I. (2024). Artificial intelligence-based myocardial infarction diagnosis: A comprehensive review. Multimed. Tools Appl..

[24] Jain S., Kumar R., Kushwah R., Saini M., Kumari S. (2025). Utilizing ensemble learning and XAI to enhance AMI prediction. Intelligent Computing and Communication Techniques.

[25] Xiong P., Lee S.M.Y., Chan G. (2022). Deep learning for detecting and locating myocardial infarction by ECG: A review. Front. Cardiovasc. Med..

[26] Jahmunah V., Ng E.Y.K., Tan R.S., Oh S.L., Acharya U.R. (2022). Explainable detection of myocardial infarction using Grad-CAM technique on ECG signals. Comput. Biol. Med..

[27] Riek N.T., Akcakaya M., Bouzid Z., Gokhale T., Helman S.M., Kraevsky K., Ji R.Q., Sejdic E., Zègre-Hemsey J.K., Martin-Gill C. (2025). ECG-Smart-Net: A deep learning architecture for occlusion myocardial infarction diagnosis. IEEE Trans. Biomed. Eng..

[28] Cao Y., Liu W., Zhang S., Xu L., Zhu B., Cui H., Geng N., Han H., Greenwald S.E. (2022). Detection and localization of myocardial infarction based on multi-scale ResNet. Front. Physiol..

[29] Xiong P., Xue Y., Zhang J., Liu M., Du H., Zhang H., Hou Z., Wang H., Liu X. (2021). Localization of myocardial infarction with multilead ECG based on DenseNet. Comput. Methods Programs Biomed..

[30] Rai H.M., Chatterjee K. (2022). Hybrid CNN-LSTM deep learning model and ensemble technique for automatic detection of myocardial infarction using big ECG data. Appl. Intell..

[31] Wang J., Guo X. (2024). Automated detection of myocardial infarction using refined LSTM/GRU. Artif. Intell. Med..

[32] Bousseljot R., Kreiseler D., Schnabel A. (2004). The PTB Diagnostic ECG Database. PhysioNet.

[33] Issa M.F., Yousry A., Tuboly G., Juhasz Z., AbuEl-Atta A.H., Selim M.M. (2023). Heartbeat classification based on single lead-II ECG using deep learning. Heliyon.

[34] Yousuf A., Hafiz R., Riaz S., Farooq M., Riaz K., Rahman M.M.U. (2024). Inferior myocardial infarction detection from lead ii of ecg: A gramian angular field-based 2d-cnn approach. IEEE Sens. Lett..

[35] Limaye H., Deshmukh V.V. (2016). ECG noise sources and various noise removal techniques: A survey. Int. J. Appl. Innov. Eng. Manag..

[36] Mallat S. (1999). A Wavelet Tour of Signal Processing.

[37] Vetterli M., Herley C. (1992). Wavelets and filter banks: Theory and design. IEEE Trans. Signal Process..

[38] McDonald A.J., Baumgaertner A.J.G., Fraser G.J., George S.E., Marsh S. (2007). Empirical mode decomposition of atmospheric wave fields. Ann. Geophys..

[39] Ge H., Chen G., Yu H., Chen H., An F. (2018). Theoretical analysis of empirical mode decomposition. Symmetry.

[40] Zamudio-Ramirez I., Saucedo-Dorantes J.J., Antonino-Daviu J.A., Osornio-Rios R.A., Dunai L. (2022). Detection of uniform gearbox wear using statistical features. IEEE Trans. Ind. Appl..

[41] Anbalagan T., Nath M.K., Anbalagan A. (2024). Detection of atrial fibrillation from ECG signal using efficient feature selection and classification. Circuits Syst. Signal Process..

[42] Aljalal M., Aldosari S.A., Molinas M., AlSharabi K., Alturki F.A. (2022). Detection of Parkinson’s disease from EEG using wavelets. Sci. Rep..

[43] Wang W., Zhao X., Luo L., Zhang P., Mo F., Chen F., Chen D., Wu F., Wang B. (2022). A Fault Diagnosis Method of Rolling Bearing Based on Attention Entropy and Adaptive Deep Kernel Extreme Learning Machine. Energies.

[44] Chanwimalueang T., Mandic D.P. (2017). Cosine Similarity Entropy: Self-Correlation-Based Complexity Analysis of Dynamical Systems. Entropy.

[45] Azami H., Faes L., Escudero J., Humeau-Heurtier A., Silva L.E. (2023). Entropy analysis of univariate biomedical signals: Review and comparison of methods. Frontiers in Entropy Across the Disciplines: Panorama of Entropy: Theory, Computation, and Applications.

[46] Li P., Liu C., Li K., Zheng D., Liu C., Hou Y. (2015). Assessing the complexity of short-term heartbeat interval series by distribution entropy. Med. Biol. Eng. Comput..

[47] Yan C., Li P., Liu C., Wang X., Yin C., Yao L. (2019). Novel gridded descriptors of poincaré plot for analyzing heartbeat interval time-series. Comput. Biol. Med..

[48] Liu X., Wang X., Zhou X., Jiang A. (2018). Appropriate use of the increment entropy for electrophysiological time series. Comput. Biol. Med..

[49] Rohila A., Sharma A. (2019). Phase entropy: A new complexity measure for heart rate variability. Physiol. Meas..

[50] Li Y., Gao P., Tang B., Yi Y., Zhang J. (2021). Double feature extraction method of ship-radiated noise signal based on slope entropy and permutation entropy. Entropy.

[51] Grivel E., Berthelot B., Colin G., Legrand P., Ibanez V. (2024). Benefits of zero-phase or linear phase filters to design multiscale entropy: Theory and application. Entropy.

[52] Kumar N., Dixit A., Vijay V. (2025). Entropy measures and their applications: A comprehensive review. arXiv.

[53] Kennedy J., Eberhart R. (1995). Particle swarm optimization. Proceedings of ICNN’95-International Conference on Neural Networks.

[54] Shami T.M., El-Saleh A.A., Alswaitti M., Al-Tashi Q., Summakieh M.A., Mirjalili S. (2022). Particle swarm optimization: A comprehensive survey. IEEE Access.

[55] Mousavi R., Eftekhari M. (2015). A new ensemble learning methodology based on hybridization of classifier ensemble selection approaches. Appl. Soft Comput..

[56] Mishra P.K., Yadav A., Pazoki M. (2018). A novel fault classification scheme for series capacitor compensated transmission line based on bagged tree ensemble classifier. IEEE Access.

[57] Polikar R. (2006). Ensemble based systems in decision making. IEEE Circuits Syst. Mag..

[58] Vapnik V.N. (1999). An overview of statistical learning theory. IEEE Trans. Neural Netw..

[59] Duda R.O., Hart P.E., Stork D.G. (2001). Pattern Classification.

[60] Martínez-Otzeta J.M., Sierra B., Lazkano E., Astigarraga A. (2006). K Nearest neighbor edition to guide classification tree learning: Motivation and experimental results. Data Mining: Theory, Methodology, Techniques, and Applications.

[61] Valafar F. (2001). Applications of neural networks in medicine. Intelligent Control Systems.

[62] Narin A. (2022). Detection of focal and non-focal epileptic seizure using continuous wavelet transform-based scalogram images and pre-trained deep neural networks. IRBM.

[63] Sadhukhan D., Pal S., Mitra M. (2018). Automated identification of myocardial infarction using harmonic phase distribution pattern of ECG data. IEEE Trans. Instrum. Meas..

[64] Diker A., Comert Z., Avci E., Velappan S. (2018). Intelligent system based on Genetic Algorithm and support vector machine for detection of myocardial infarction from ECG signals. SIU 2018.

[65] Lui H.W., Chow K.L. (2018). Multiclass classification of myocardial infarction with convolutional and recurrent neural networks for portable ECG devices. Inform. Med. Unlocked.

[66] Feng K., Pi X., Liu H., Sun K. (2019). Myocardial infarction classification based on convolutional neural network and recurrent neural network. Appl. Sci..

[67] Sun Q., Li J., Liang C., Liu R., Pang J., Chen Y., Wang C. (2025). A multi-lead group network for myocardial infarction detection and localization based on clinical knowledge-driven and dynamic-static feature fusion. Expert. Syst. Appl..

[68] Sopic D., Aminifar A., Aminifar A., Atienza D. (2018). Real-time event-driven classification technique for early detection and prevention of myocardial infarction on wearable systems. IEEE Trans. Biomed. Circuits Syst..

[69] Shahnawaz M.B., Dawood H. (2021). An Effective Deep Learning Model for Automated Detection of Myocardial Infarction Based on Ultrashort-Term Heart Rate Variability Analysis. Math. Probl. Eng..

[70] Jian J.-Z., Ger T.-R., Lai H.-H., Ku C.-M., Chen C.-A., Abu P.A.R., Chen S.-L. (2021). Detection of myocardial infarction using ECG and multi-scale feature concatenate. Sensors.

[71] Sheth K.A., Upreti C., Prusty M.R., Satapathy S.K., Mishra S., Cho S.-B. (2024). Time-frequency transformation integrated with a lightweight convolutional neural network for detection of myocardial infarction. BMC Med. Imaging.

